# Apolipoprotein(a) is the Product of a Pseudogene: Implications for the Pathophysiology of Lipoprotein(a)

**DOI:** 10.7759/cureus.2715

**Published:** 2018-05-31

**Authors:** Gregory D Sloop, Gheorghe Pop, Joseph J Weidman, John A St. Cyr

**Affiliations:** 1 Pathology, Idaho College of Osteopathic Medicine, Meridian, USA; 2 Cardiology, Radboud University Nijmegen Medical Center, Nijmegen, The Netherlands, Nijmegen, NLD; 3 N/A, Independent Researcher; 4 Research and Development, Jacqmar, Inc., Minneapolis, USA

**Keywords:** atherothrombosis, apolipoprotein(a), lipoprotein a, pseudogene, blood viscosity

## Abstract

Apolipoprotein(a) [apo(a)] is an apolipoprotein unique to lipoprotein(a) [Lp(a)]. Although it has no known function, Lp(a) is a risk factor for accelerated atherothrombosis. We hypothesize that LPA, the gene which encodes apo(a), is a heretofore unrecognized unprocessed pseudogene created by duplication of PLG, the gene which encodes plasminogen. Unprocessed pseudogenes are genes which were created by duplication of functional genes and subsequently lost function after acquiring various mutations. This hypothesis explains many of the unusual features of Lp(a) and apo(a). Also, this hypothesis has implications for the therapy of elevated Lp(a) and atherothrombosis theory. Because apo(a) is functionless, the diseases associated with elevated levels of Lp(a) are due to its impact on blood viscosity.

## Introduction and background

Lipoprotein(a) and apolipoprotein(a)

Lipoprotein(a) [Lp(a)] is a particle composed of a core which is indistinguishable from that of low density lipoprotein (LDL) and a single molecule of apolipoprotein(a) [apo(a)]. Lp(a) is clinically significant because it is a risk factor for accelerated atherothrombosis as well as arterial and venous thrombosis. Lp(a) and apo(a) have several unusual features. After decades of study, no physiologic function has been definitively attributed to either. Plasma concentrations of Lp(a) vary by three orders of magnitude, from undetectable to > 200 mg/dl [1]. This degree of variation is very unusual for an analyte present in mg/dl concentrations. The extreme variation in molecular weight of apo(a) is unique [2]. This is due to variation in the number of kringle IV-like domains in the protein. These domains are important in protein-protein interactions, and are present in several different proteins, including plasminogen. The similarities between apo(a) and plasminogen are widely-recognized. Apo(a) possesses between 15 to 43 copies of this domain [1]. This variation reflects differences in LPA, the gene which encodes apo(a). There are at least 34 alleles of this gene, including a null allele which does not produce protein. Because these alleles are present in roughly similar frequencies, heterozygosity (the percentage of individuals who are heterozygous at a particular locus) for LPA is extraordinarily high. In a study of multiple populations, the average heterozygosity at the LPA locus was 94%, the highest reported up to that time [3].

Pseudogenes

Pseudogenes have been defined as copies of protein-encoding genes that are thought to no longer have the same functional product as their parental gene but still share significant sequence similarity [4]. LPA has significant homology to PLG, the gene which encodes plasminogen, but has a 27 nucleotide deletion in the protease domain, among several other differences [vide infra]. Thus, apo(a) can be considered a defective form of plasminogen which qualifies LPA as a pseudogene. In humans, it is estimated that there are approximately 16,881 pseudogenes, compared to 20,687 genes which encode functional proteins. Approximately 168 pseudogenes express peptides or proteins [5].

Three types of pseudogenes are recognized: processed, unprocessed, and unitary. Processed pseudogenes are created when complementary DNA (cDNA) is synthesized from mRNA and then inserted into a DNA strand. Because a promoter is not present in cDNA, processed pseudogenes are functionless. It is estimated that humans have approximately 8,000 processed pseudogenes [6]. 

**Figure 1:**
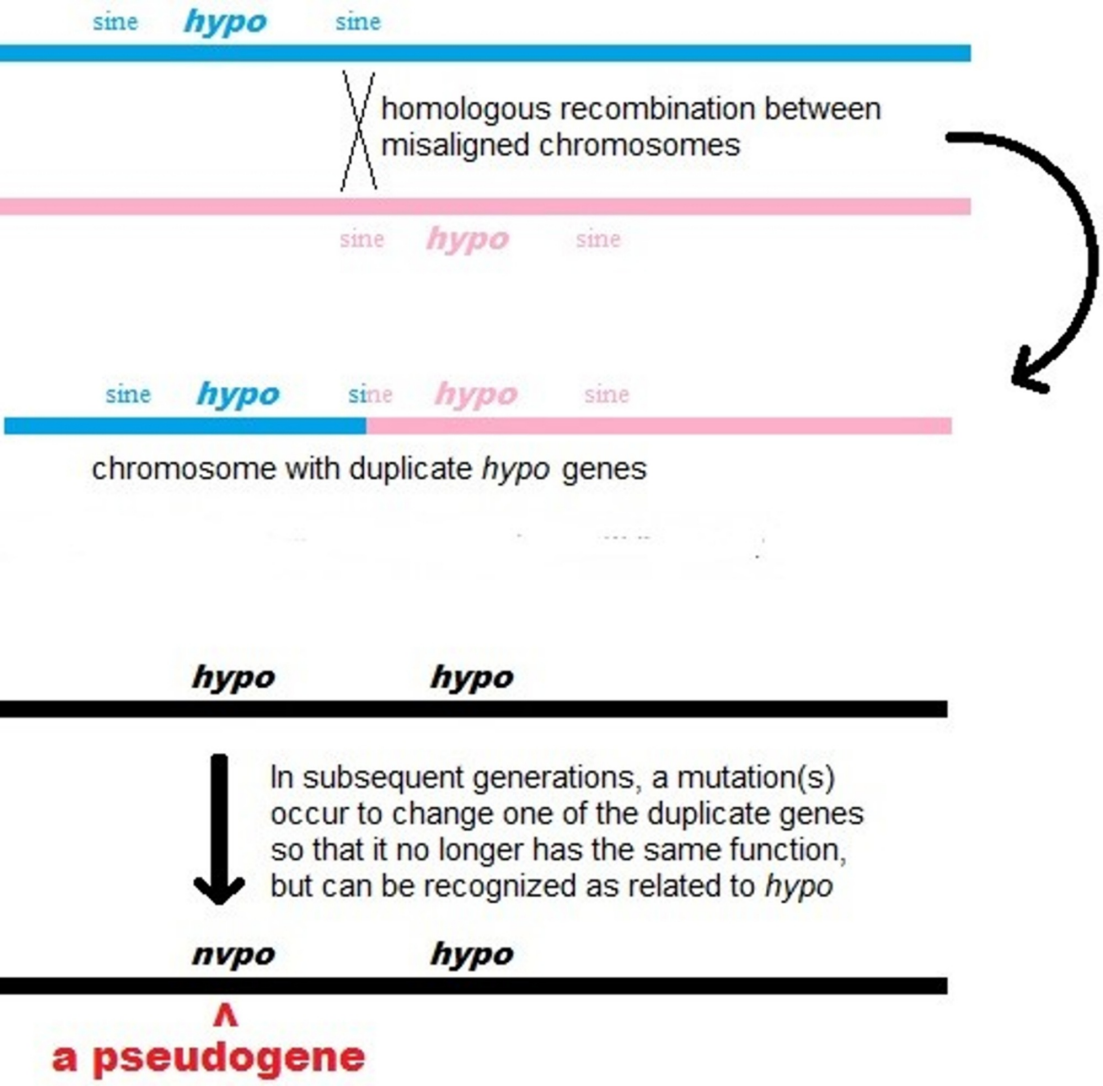
Generation of an unprocessed pseudogene by unequal recombination of homologous genes due to misalignment In LPA, the gene which encodes apolipoprotein(a), two Alu elements, the most common type of SINE (short interspersed nuclear element), are present in the untranslated 5’ region. (Modified from original artwork by Dennis Pietras. From Wikimedia Commons.)

Unprocessed pseudogenes are created by unequal recombination during crossover in meiosis (Figure 1).

This occurs when misalignment of homologous genes causes one chromosome to have two copies of the gene and the other none following DNA replication. Misalignment happens when two DNA strands bind at the wrong conserved repetitive sequence. Conserved repetitive sequences such as SINEs (short interspersed nuclear elements) are DNA sequences which are repeated multiple times throughout the genome. The most common SINEs are Alu elements, a family of DNA sequences approximately 300 base pairs long which are repeated over one million times and comprise approximately 10% of the human genome [7].

The high prevalence of conserved repetitive sequences explains why duplicate genes arise at a “very high” rate, an average of 0.01 per gene per million years. Because duplicate genes are generally assumed to be functionally redundant, their likeliest evolutionary outcome is to accumulate mutations, creating a pseudogene. Alternatively, duplicate genes may persist in an effectively neutral or beneficial manner [8]. Both outcomes are seen in the alpha-like globin cluster on chromosome 16 and the beta-like globin cluster on chromosome 11. Both clusters were formed by multiple duplication events. The alpha globin cluster consists of five functional genes and two unprocessed pseudogenes, HBAP1 and HBZP1. The beta globin cluster consists of five coding genes and one unprocessed pseudogene, HBBP1. The functional genes in both clusters are sequentially expressed in development in the order they evolved, resulting in distinct hemoglobins during the embryonic, fetal, and adult stages, an example of ontogeny recapitulating phylogeny.

Recently, functionality has been attributed to some genes classified as pseudogenes. The best documented example is said to be XIST, or X inactive specific transcript, which has sequence homology to a chicken gene called LNX3 [9]. XIST is transcribed but not translated. It encodes an RNA which silences transcription of many genes on one X chromosome in females, an example of a long noncoding RNA (lncRNA) modifying gene expression. The crux of the argument that XIST is a pseudogene is that the gene does not express protein and is surrounded by pseudogenes [10]. A mutation which prevents a gene from being normally expressed creates a unitary pseudogene. XIST is surrounded by two unrelated processed pseudogenes, FXYD6P3, which is one of three pseudogenes derived from the gene which encodes FXYD domain-containing ion transport regulator 6, a sodium transport channel regulator, and SEPHS1P4, one of four pseudogenes derived from the gene encoding selenophosphate synthetase 1.

Instead of “pseudogenization,” Elisaphenko et al. demonstrated that XIST arose de novo from four exons from LNX3 and six exons consisting of short tandem repeats derived from transposons [11]. It is the latter which are necessary for transcriptional silencing of the X chromosome. A mutation in the start codon caused loss of protein expression before new exons imparted function to XIST, creating a new gene. Thus, the crucial contribution of LNX3 was the promoter region, which is among the most highly conserved regions of XIST, not the exons that encode a protein which is obviously nonessential.

The process which produced XIST is the evolution of a unitary pseudogene into one with a function. This is fundamentally different from the mutations which created the processed pseudogenes which surround XIST, the unprocessed pseudogenes in the alpha and beta globin clusters, or the unitary pseudogene precursor to XIST. It is debatable if the mere presence of a nucleotide sequence which is homologous to one in a gene which produces a protein constitutes a pseudogene because expression of a protein is not necessary for a gene to be functional. XIST is an example of a gene whose function is mediated by lncRNA, not a protein. The presence of processed pseudogenes adjacent to XIST may simply be a consequence of their prevalence.

It has been suggested that antisense transcription can impart functionality to a pseudogene. Transcription of both strands of DNA, a process known as convergent transcription, is extremely unlikely because RNA polymerase II complexes cannot bypass each other in vitro, and transcription ceases when they collide [12]. It has also been suggested that mRNA encoded by a “pseudogene” could be functional by binding to microRNA and preventing it from silencing other mRNAs. If such a gene is present in germline DNA, it can be argued that it is a product of evolution, and the gene is not a pseudogene. The label “pseudogene” was originally applied to genes which were thought to be nonfunctional. To apply this term to genes which have a function is misleading and distorts the meaning of a useful term.

## Review

The authors hypothesize that LPA is a heretofore unrecognized unprocessed pseudogene. LPA arose in a primate ancestor from duplication of PLG, the gene which encodes plasminogen. The 5’ untranslated regions of LPA and PLG are nearly identical, and contain two Alu elements [13]. After this duplication event, the gene subsequently underwent deletions of kringles I-III as well as deletions in the sequences encoding the protease domain and preactivation peptide. Also, kringle IV was duplicated a highly variable number of times. Because of these mutations, apo(a) has no fibrinolytic activity [1].

Gene sequences with function tend to be evolutionarily conserved while nonfunctional sequences diverge because of the accumulation of duplications, deletions and other mutations [14]. The accumulation of mutations and large variation in the number of kringle IV sequences strongly suggests that LPA is a nonfunctional gene. It would be surprising if a gene with so many common alleles expressed a protein with a necessary function. Not surprisingly, humans with loss of function mutations at the LPA locus are healthy [15].

The apo(a)-plasminogen gene cluster

**Figure 2:**
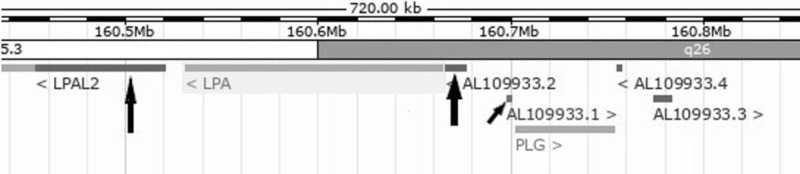
Chromosome 6q25.3-6q26 The genes LPAL2 (lipoprotein(a)-like 2), an unprocessed pseudogene, LPA, and PLG, the gene encoding plasminogen, are clustered in this region. Pseudogenes are present on both sides of LPA (large arrows). A long intergenic noncoding RNA (lincRNA) is indicated by the small arrow. (Courtesy of Ensembl 92. http://uswest.ensembl.org/Homo_sapiens/Location/Overview?db=core;g=ENSG00000198670;r=6:160237877-160957878)

LPA is surrounded by unprocessed pseudogenes (Figure 2).

One, named LPAL2, is homologous to LPA, but all transcripts are truncated and eliminated by nonsense-mediated decay, a surveillance pathway which eliminates mRNAs that have premature stop codons. The second pseudogene is designated AL109933.2. This gene contains a plasminogen tail-like sequence with a frameshift mutation resulting in a stop codon. A second mutation which destroys a consensus splicing site is found in a large intron separating the exon coding for the leader from the one encoding the tail-like sequences [16].

Finally, a long intergenic noncoding RNA (lincRNA) is present between AL109933.2 and PLG. Thus, PLG has undergone at least three separate duplications, creating three unprocessed pseudogenes. PLG, LPA, LPAL2, AL109933.2, and the gene encoding the lincRNA comprise the apo(a)-plasminogen gene cluster.

Lp(a) is a modified LDL

Lp(a) does not have a triacylglycerol-rich precursor, unlike LDL [17]. Instead, Lp(a) derives from the disulfide linkage of apo(a) to apolipoprotein B-100 (apoB-100) of pre-existing LDL. ApoB-100 undergoes conformational changes which uncover new epitopes in the transition from very low density lipoprotein (VLDL) to LDL [18]. Because apoB-100 forms a belt around the lipid core of a particle, the decrease in circumference accompanying the transition from VLDL to LDL may expose these new epitopes. Alternatively, loss of the other apolipoproteins in VLDL could cause the conformational changes. These conformational changes could explain why apo(a) binds to LDL and not VLDL. Koschinsky, et al. demonstrated that the disulfide linkage between apoB-100 and apo(a) occurs extracellularly in a two-step process. First, a strong noncovalent bond is formed between the two molecules, which is followed by the covalent disulfide linkage [19].

Because Lp(a) is modified LDL, changes in the concentration of Lp(a) parallel changes in the concentration of LDL. Sandholzer, et al. found that concentrations of both LDL and Lp(a) were very low in subjects with lipoprotein lipase deficiency and Type III hyperlipidemia [17]. Reductions of Lp(a) strongly correlated with reductions of LDL and apolipoprotein B in trials of proprotein convertase subtilisin/kexin type 9 (PCSK9) inhibitors [20]. Statin therapy reduced levels of both LDL and Lp(a) in patients with heterozygous familial hypercholesterolemia [21]. Drugs which increase the number of LDL receptors (LDLR) such as statins and PCSK9 inhibitors are more effective in reducing LDL levels than Lp(a) levels because Lp(a) competes poorly with LDL for binding to LDLR [22]. Additionally, Lp(a) levels are elevated in subjects with familial hypercholesterolemia [23].

Implications for atherothrombosis theory and therapy

The true nature of LPA as a pseudogene may have escaped notice because the overwhelming majority of pseudogenes do not express protein. Perhaps more importantly, uncertainty about the pathogenesis of atherothrombosis made it impossible to eliminate an unrecognized function for Lp(a) and apo(a). Even the most optimistic proponent of lipid or inflammatory theory must recognize that those theories are inadequate to explain important phenomena such as the protection afforded by premenopausal status or increased levels of high density lipoprotein (HDL), or the circadian variation in atherothrombotic events. We have recently reviewed the shortcomings of mainstream atherothrombosis theory [24]. 

Accumulation of Lp(a) in the arterial intima is thought to accelerate atherothrombosis, possibly because of putative residual function of apo(a). Accumulation of Lp(a) (or LDL) in the arterial wall cannot be the cause of accelerated atherothrombosis for several reasons. All plasma macromolecules, including LDL and Lp(a), are present throughout the arterial intima in concentrations directly related to their molecular weight and plasma concentration [25]. Macromolecular transport across the venous endothelium is even greater than across the arterial endothelium [26], and veins do not develop atherothrombosis. Therefore, the presence of LDL or Lp(a) in the arterial intima cannot explain the characteristic localization of atherosclerotic plaques to areas of low shear and changing arterial geometry. The glycosaminoglycans, fibronectin, and collagen necessary to retain LDL or Lp(a) are present throughout the arterial intima [25]. The quantity of these lipoprotein-binding molecules is increased in diffuse intimal thickening, a universal change in the arteries of adults [25]. The increased trapping of LDL and Lp(a) in atherosclerotic plaques is simply a consequence of the great amount of collagen and proteoglycans in plaques. Type Vc atheroscleroic plaques have little or no extra-celluar lipid, and consist of collagen and proteoglycans [27]. In the experience of one author (GDS), this is the most common type of plaque, particularly since the widespread use of statins. 

Further, a mechanism is present in the intima to eliminate plasma constituents which enter the subendothelial space. This should not be surprising because their presence is physiologic. Dendritic cells, a normal component of the intima, constitutively sample the aqueous environment of the subendothelial space by macropinocytosis. Dendritic cells can pinocytose 100x their volume per hour in vitro. Molecules which cannot be catabolized by lysosomal enzymes such as cholesterol are packaged in exosomes which allows them to circulate to the liver for catabolism. Dendritic cells assume the morphology of foam cells because their great capacity for pinocytosis allows them to accumulate cholesterol faster than it can be eliminated. The ability to eliminate cholesterol allows fatty streaks, the putative precursor to the atherosclerotic plaque, to routinely resolve without pathologic sequelae [28]. We have reviewed this pathway in reference [29]. 

The notion that the accumulation of Lp(a), LDL, or cholesterol in the intima is atherogenic is an example of the “fallacy of the simple explanation,” which states that the appeal of simplicity causes acceptance of simplistic and incorrect explanations. The words of HL Mencken should always be remembered: “For every complex problem, there is a solution that is simple, direct, and wrong” [24].

Instead, we believe that the accelerated atherothrombosis associated with elevated Lp(a) (and LDL) is due to increased blood viscosity in areas of low shear rate [29]. These develop in areas of flow separation which occur naturally in areas of changing vascular geometry. In these areas, Lp(a) and LDL foster progressive erythrocyte aggregation which increases blood viscosity. Unremitting positive feedback may develop whereby increased blood viscosity allows further erythrocyte aggregation, which further increases viscosity and slows flow, allowing further aggregation, etc. Areas of sluggish blood flow are predisposed to thrombosis as noted by the German pathologist Rudolph Virchow in the 19th century. Atherosclerotic plaques develop from the organization of mural thrombi, as originally proposed by the Welsh pathologist John Duguid in the mid-twentieth century [29]. 

The association of elevated Lp(a) with arterial thrombosis [30] and venous thromboembolism [31] is more consistent with an effect on blood viscosity than putative pathologic effects caused by its accumulation in a vessel wall. Venous thromboembolism associated with elevated levels of Lp(a) has even been noted in children [32].

The most common risk factors for accelerated atherothrombosis are associated with increased blood viscosity [29, 33-35]. Duguid’s thrombogenic hypothesis explains why diverse risk factors produce the same lesion, the atherosclerotic plaque. It also provides insight into the protection against atherothrombosis afforded by HDL, which decreases blood viscosity [36], the failure of cholesteryl ester transfer protein inhibitors [37] and the excess cardiovascular mortality caused by consumption of the Western diet and trans fats [38]. Our study of blood viscosity has also provided insight into the cause of several anemias, including those associated with heart failure [39], chronic disease and inflammation, and hemolytic anemias [40].

Lipoprotein apheresis is the most effective therapy for reducing Lp(a) levels. Most methods remove all apolipoprotein B-containing lipoproteins. This procedure has been shown to decrease plasma viscosity [41-42]. In areas where therapeutic apheresis is not available, therapeutic phlebotomy and blood donation are alternative means of lowering blood viscosity. These interventions improve blood viscosity by replacing older, stiffer erythrocytes with fresh deformable ones. Blood donation has two further advantages: it helps recipients of the donation, and mobile blood collection vehicles can extend the therapy to underserved areas. A high-risk population such as those with elevated plasma levels of Lp(a) without clinical disease could be an ideal population for a prospective study of blood donation on atherothrombosis. The fact that Lp(a) has no function suggests that oligonucleotide antisense therapy may be safe if the oligonucleotide does not cross-react with other nucleotides.

The existence of Lp(a) is a consequence of a chance event, the mistake in homologous recombination which created LPA. It did not result from an evolutionary development which conferred a competitive advantage. The fact that apo(a) is the product of a pseudogene explains many of the unusual properties of Lp(a). No function is to be expected in the product of a pseudogene. The extreme heterogeneity in the size of apo(a) is due to the fact that there is no constraint on the number of mutations a pseudogene can acquire. With the exception of a premature stop codon, changes in a gene, such as the number of kringle sequences, will be reflected in the protein encoded by that gene. The extreme variation in plasma concentrations of Lp(a) at both ends of its range is also explained. Obviously, absence of a functionless particle is not disadvantageous. The variability in the size of apo(a) also plays a role in determining plasma concentrations because there is an inverse correlation between apo(a) size and plasma concentration of Lp(a). The number of kringle IV repeats accounts for 69% of variance in plasma concentrations of Lp(a). Overall, 91% of variance in plasma concentrations of Lp(a) is determined genetically [43]. The fact that there is no constraint on the accumulation of mutations also explains the extraordinary degree of heterozygosity in apo(a) genotype. Because mutations in LPA have no impact on reproductive fitness, there is no selective pressure acting to eliminate an allele from the gene pool. Boerwinkle et al. even observed the de novo generation of a new allele during the course of their study, an event which occurred once in 376 meioses [43]. Presumably, expression of apo(a) is controlled by the same regulatory elements as plasminogen. Plasma concentrations of plasminogen vary little or none over 24 hours [44]. This explains why changes in lifestyle have little impact on plasma concentrations of Lp(a).

## Conclusions

The insight that apo(a) is the product of a pseudogene clarifies much of the mystery surrounding Lp(a): its lack of function, its remarkable variation in plasma concentration, and why its absence is not associated with ill health. It explains the wide variation in the molecular weight of apo(a) and the extreme heterogeneity at the LPA locus. These characteristics are inconsistent with a functional entity under homeostatic control. This insight also clarifies the role of Lp(a) in accelerated atherothrombosis. Lack of function is one reason why accumulation of apo(a) in the arterial wall does not cause the accelerated atherothrombosis associated with elevated levels of Lp(a). Rather, Lp(a) (and LDL) accelerate atherothrombosis by increasing blood viscosity. The most effective intervention for elevated levels of Lp(a), therapeutic apheresis, works by decreasing blood viscosity. In areas where this intervention is not available, therapeutic phlebotomy or blood donation are alternatives.
